# Natural Transmission of Helicobacter saguini Causes Multigenerational Inflammatory Bowel Disease in C57/129 IL-10^−/−^ Mice

**DOI:** 10.1128/mSphere.00011-20

**Published:** 2020-03-25

**Authors:** Anthony Mannion, Zeli Shen, Yan Feng, Dylan Puglisi, Sureshkumar Muthupalani, Mark T. Whary, James G. Fox

**Affiliations:** a Division of Comparative Medicine, Massachusetts Institute of Technology, Cambridge, Massachusetts, USA; University of Michigan—Ann Arbor

**Keywords:** DNA damage, *Helicobacter*, cotton-top tamarins, germfree IL-10^−/−^ mice, inflammatory bowel disease, microbiome, microevolutions, multigenerational, whole-genome sequencing

## Abstract

Cotton-top tamarins (CTTs) are an ideal model of human inflammatory bowel disease (IBD) because these animals develop multigenerational, lower bowel cancer. We previously isolated and characterized a novel enterohepatic *Helicobacter* species, Helicobacter saguini, from CTTs with IBD and documented that H. saguini infection in germfree C57BL IL-10^−/−^ mice recapitulates IBD, suggesting that *H. saguini* influences IBD etiopathogenesis. In this study, we utilized a germfree IL-10^−/−^ model to illustrate that *H. saguini* infection can naturally transmit and infect four generations and cause significant intestinal inflammatory pathology. Additionally, whole-genome sequencing of representative *H. saguini* isolates from each generation of IL-10^−/−^ mice revealed gene mutations suggestive of multigenerational evolution. Overall, these results support that specific bacterial species with pathogenic potential, like *H. saguini*, are transmissible microorganisms in the etiopathogenesis of IBD in CTTs and reinforces the importance of specific microbiota in the pathogenesis of IBD in humans.

**IMPORTANCE** While family history is a significant risk factor for developing inflammatory bowel disease (IBD), it is unclear whether the microbiome from parents is a transmissible influence on disease in their offspring. Furthermore, it is unknown whether IBD-associated microbes undergo genomic adaptations during multigenerational transmission and chronic colonization in their hosts. Herein, we show that a single bacterial species, Helicobacter saguini, isolated from a nonhuman primate species with familial IBD, is transmissible from parent to offspring in germfree IL-10^−/−^ mice and causes multigenerational IBD. Additionally, whole-genome sequence analysis of *H. saguini* isolated from different mouse generations identified microevolutions in environmental interaction, nutrient metabolism, and virulence factor genes that suggest that multigenerational transmission may promote adaptations related to colonization and survival in new hosts and chronic inflammatory environments. The findings from our study highlight the importance of specific bacterial species with pathogenic potential, like *H. saguini*, as transmissible microorganisms in the etiopathogenesis of IBD.

## Abstract (precis)

While family history is a significant risk factor for developing inflammatory bowel disease (IBD), it is unclear whether the microbiome from parents is a transmissible influence on disease in their offspring. Furthermore, it is unknown whether IBD-associated microbes undergo genomic adaptations during multigenerational transmission and chronic colonization in their hosts. Herein, we show that a single bacterial species, Helicobacter saguini, isolated from a nonhuman primate species with familial IBD, is transmissible from parent to offspring in germfree IL-10^−/−^ mice and causes multigenerational IBD. Additionally, whole-genome sequence analysis of *H. saguini* isolated from different mouse generations identified microevolutions in environmental interaction, nutrient metabolism, and virulence factor genes that suggest that multigenerational transmission may promote adaptations related to colonization and survival in new hosts and chronic inflammatory environments. The findings from our study highlight the importance of specific bacterial species with pathogenic potential, like *H. saguini*, as transmissible microorganisms in the etiopathogenesis of IBD.

## OBSERVATION

While remaining idiopathic, inflammatory bowel disease (IBD) is the multifactorial result of genetic, environmental, and microbial interactions. Family history is the greatest risk factor (1, 2). The concordance rate of IBD in monozygotic twins is <50%, indicating incomplete genetic penetrance and reinforcing the importance of shared environmental factors, including the microbiome (3–6). Cotton-top tamarins (Saguinus oedipus) (CTTs) are nonhuman primates that, when in captivity, develop multigenerational, idiopathic IBD that progresses to colon cancer (7–10). These animals are considered an ideal model for IBD due to their clinical and histopathological similarities with human IBD; however, they are no longer used because they have been placed on the endangered species list. Interestingly, CTTs are more likely to develop colitis when cohoused in a colony with endemic IBD than when reared apart, suggesting transmission of specific microorganisms, which could be etiologically important (7). Our lab isolated Helicobacter saguini, a novel enterohepatic *Helicobacter* species (EHS), from the feces and colons of captive CTTs with IBD (9). Given that EHS infection has been associated with IBD in human patients and macaque species (11, 12) and that other EHS experimentally induce IBD in murine models (13), we hypothesized that H. saguini infection is associated with IBD in CTTs. Recently, we demonstrated that mono-associated *H. saguini* infection in germfree IL-10^−/−^ mice caused typhlocolitis and argued that *H. saguini* contributes to IBD in CTTs (14). *H. saguini*, however, was unable to colonize specific-pathogen-free (SPF) IL-10^−/−^ mice (14). In this study, we used the germfree IL-10^−/−^ mouse model to test the hypothesis that *H. saguini* infection in captive CTT colonies is naturally transmitted through successive generations resulting in colitis in infected animals. Additionally, we studied how multigenerational *H. saguini* colonization yielded microevolutions in its genome that may have promoted chronic colonization and the sustained ability of *H. saguini* to induce pathogenicity.

## 

### Experimental results.

**FIG 1:**
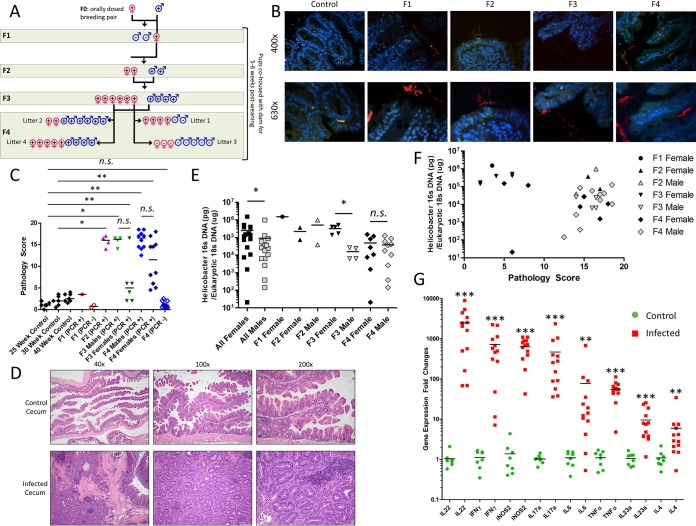
(A) Diagram showing experimental design for multigenerational infection study in germfree IL-10^−/−^ mice. A male (♂) and female (♀) breeding pair (F0 generation) were orally dosed with *H. saguini* and then bred to produce the F1 generation. The infected F1 female was bred with the F0 male to yield F2, and brother-sister mating produced the sequential F3 and F4 generations. Pups remained with the dams for 5 to 6 weeks after weaning. Colonization with *H. saguini* was confirmed by PCR analysis of feces using the *Helicobacter* genus-specific primers and shown by a plus or minus sign within the gender symbol to designate which animals were positive or negative for infection, respectively. Mice were euthanized and necropsied from 5 to 40 weeks old (average ages of the mice in the different generations follow: F1, ∼40 weeks old; F2, ∼30 weeks old; F3, ∼25 weeks old; F4, ∼25 weeks old). Three cohorts of mice were included as age-matched controls (not shown in the diagram). (B) Fluorescence *in situ* hybridization (FISH) using a *Helicobacter* genus-specific probe in mouse ceca from representative mice of the different generations. *H. saguini* cells are labeled in red. 4′,6′-Diamidino-2-phenylindole (DAPI) stained the nuclei blue. (C) Histopathological scores for the ceca of germfree IL-10^−/−^ mice from F1, F2, F3, and F4 generations compared to age-matched controls. Mice were separated based on sex and whether they were infected with *H. saguini* as determined by PCR [infected (PCR +) or not infected (PCR -)]. Values that are significantly different are indicated by bars and asterisks as follows: *, *P* value of <0.05; **, *P* value of <0.01. Values that are not significantly different (*n.s.*) are indicated. (D) Representative hematoxylin and eosin (H&E) staining images of cecum from *H. saguini*-infected IL-10^−/−^ mouse from F4 generations compared to 40-week-old control mouse. *H. saguini* infection caused severe inflammation and architectural malformations to epithelial, mucosal, and submucosal layers of the cecum, not observed in the uninfected control mice. Images were taken at 40×, 100×, and ×200 magnifications, and bars represent 500, 200, and 100 μm distance, respectively. (E) Comparison of the colonization loads of *H. saguini* in feces from male and female mice from the F1, F2, F3, and F4 generations. *, *P* value of <0.05; *n.s.*, not significant. (F) Histopathology scores in ceca versus colonization loads of *H. saguini* in feces from male and female mice from the F1, F2, F3, and F4 generations. (G) Inflammatory gene expression in cecal tissue in a representative litter from the F4 generation. Expression of each inflammatory gene was significantly greater in infected mice than in age-matched control mice (**, *P* value < 0.01; ***, *P* value < 0.001; *n.s.*, not significant). The data are presented as the fold change compared to the mean gene expression of glyceraldehyde-3-phosphate dehydrogenase (GAPDH).

To test whether *H. saguini* infection can be naturally transmitted to multiple generations, a male and female breeding pair (F0 generation) were orally dosed and colonized with *H. saguini*. Breeding of F0 yielded three pups (F1), one of which naturally acquired infection after being cohoused with its dam (Fig. 1A). Breeding of the infected F1 female with the F0 male yielded F2, and subsequent brother-sister mating produced F3 followed by F4 that also naturally acquired *H. saguini* infection (Fig. 1A). For all generations, pups remained cohoused with their dams for 5 to 6 weeks after weaning to promote natural transmission of *H. saguini* infection. Infected mice remained *H. saguini* PCR positive at necropsy. Fluorescence *in situ* hybridization (FISH) of the ceca of representative mice from each generation demonstrated that *H. saguini* localized at the mucosal surface, while no bacteria were present in the age-matched control mice (Fig. 1B). These data indicate that *H. saguini* infection chronically colonized germfree IL-10^−/−^ mice for up to ∼40 weeks and that infection with *H. saguini* can be naturally transmitted from dams to offspring over four successive generations, likely via fecal-oral horizontal transmission.

Mice PCR positive for *H. saguini* infection in all four generations developed significant inflammatory pathology in the cecum and colon compared to their respective age-matched controls (Fig. 1C; see also Fig. S1A in the supplemental material), except generation F1 in which only 1/3 mice were positive for infection. Litter 3 from generation F4, which failed to naturally acquire infection, did not have statistically different histology scores compared to those of the controls. Pathology in the cecum and colon was characterized by marked infiltration of inflammatory cells in the mucosa and submucosa along with dysplasia, hyperplasia, and epithelial and crypt defects (Fig. 1D and Fig. S1B). Interestingly, male mice from the F3 and F4 generations had considerably higher median cecum and colon pathology scores compared to their female counterparts, although the difference did not reach statistical significance (Fig. 1C and Fig. S1A). Fecal colonization of *H. saguini* was statistically higher in females than in males in the F3 generation and when all generations were pooled (Fig. 1E). Furthermore, we observed that mice colonized with ≥∼10^5^ copies of *H. saguini* have cecum and colon pathology scores of <10 and were primarily females, while the opposite trend was seen for males (Fig. 1F and Fig. S1C). This suggests that females may tolerate higher colonization densities and elicit less robust pathology responses to *H. saguini* compared to males in this model.

10.1128/mSphere.00011-20.1 FIG S1 (A) Histopathological scores for the colons of germfree IL-10^−/−^ mice from F1, F2, F3, and F4 generations compared to age-matched controls. Mice were separated based on sex and whether they were infected with *H. saguini* as determined by PCR (infected, PCR +; noninfected, PCR -). *, *P* value < 0.05; **, *P* value < 0.01; *n.s.*, not significant. (B) Representative H&E staining images of a colon from a *H. saguini*-infected IL-10^−/−^ mouse from F4 generation compared to a40-week-old control mouse. *H. saguini* infection caused severe inflammation and architectural malformations to epithelial, mucosal, and submucosal layers of the colon, not observed in the uninfected control mice. Images were taken at 40×, 100×, and 200× magnifications, and bars represent 500, 200, and 100 μm distance, respectively. (C) Histopathology scores in colon versus colonization loads of *H. saguini* in feces of male and female mice from the F1, F2, F3, and F4 generations. (D) Representative immunohistochemistry images of cecum stained for γ-H2AX (green) and nuclei (DAPI; blue) from two *H. saguini*-infected mice from the F4 generation and two age-matched control mice. (E) Quantification of γ-H2AX-positive cells located in crypt epithelium in a representative litter from the F4 generation compared to age-matched control mice. **, *P* value of < 0.01. Download FIG S1, TIF file, 2.6 MB. Copyright © 2020 Mannion et al. 2020 Mannion et al. This content is distributed under the terms of the Creative Commons Attribution 4.0 International license.

The levels of inflammatory cytokines for interleukin 22 (IL-22), gamma interferon (IFN-γ), inducible nitric oxide synthase 2 (iNOS2), IL-17a, IL-6, tumor necrosis factor alpha (TNF-α), IL-23a, and IL-4 expressed in the ceca of a representative F4 litter were also significantly higher than those for age-matched controls (Fig. 1G). Immunohistochemistry was performed for the DNA histone modification, γ-H2AX, a marker for DNA damage (15) and apoptotic cells (16). *H. saguini*-infected mice from a representative F4 litter demonstrated significantly higher levels of γ-H2AX in the cecum compared to age-matched controls (Fig. S1D and E). These results indicated that multigenerational, chronic *H. saguini* infection induces inflammatory gene pathways as well as DNA damage and cell death.

**FIG 2:**
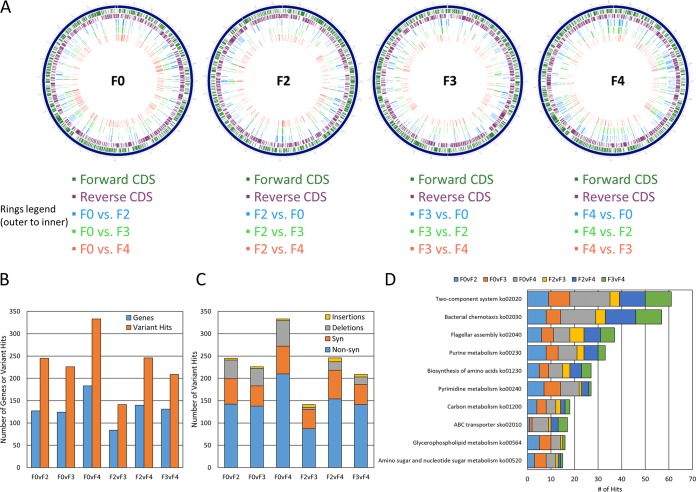
(A) Graphical circular maps of chromosomes from *H. saguini* F0, F2, F3, and F4 isolates. The rings from the outermost ring to the innermost ring designate forward protein coding sequences (CDS), reverse CDS, and locations of gene variants between generational pairwise comparisons. (B) Number of variant genes or total variant hits detected in F0 versus F2, F3, and F4 isolate genomes. (C) Number of synonymous, nonsynonymous, insertion, and deletion variants detected in F0 versus F2, F3, and F4 isolate genomes. (D) KEGG automatic annotation server (KAAS) analysis to determine the top 10 most frequent KEGG (Kyoto Encyclopedia of Genes and Genomes) metabolic/functional pathways associated with the variant genes detected for F0 versus F2, F3, and F4 isolate genomes.

Genomic microevolutions have been reported for the gastric carcinogen Helicobacter pylori during chronic host colonization (17, 18) and transmission to family members (19–21) as well as for the gastroenteritis-causing pathogen Campylobacter jejuni (a close relative to EHS) during adaption to new host species (22–26). However, whether microbes associated with IBD undergo genomic microevolution within their hosts is unknown. We sought to address this question by performing comparative genomic analyses between multigenerational *H. saguini* isolates. Representative *H. saguini* isolates were cultured from F0, F2, F3, and F4 mice. Although the mouse from the F1 generation had high *H. saguini* colonization levels (Fig. 1E), an isolate could not be recovered possibly because the organism did not remain viable/culturable after sample collection and frozen storage. Genomes for F0 to F4 isolates were sequenced using Illumina MiSeq and PacBio for high-quality *de novo* contig assemblies (see Tables S1 and S2 in the supplemental material). Gross rearrangements in chromosomal regions were not detected in the isolates. Variant analysis identified synonymous, nonsynonymous, insertion, and deletion mutational differences between the multigenerational genomes (Table S3) that occurred throughout the chromosome (Fig. 2A). Overall, the number of variants increased in a generational manner (Fig. 2B and C).

10.1128/mSphere.00011-20.2 TABLE S1 Summary statistics for multigenerational *H. saguini* genome sequencing. Download Table S1, DOCX file, 0.01 MB. Copyright © 2020 Mannion et al. 2020 Mannion et al. This content is distributed under the terms of the Creative Commons Attribution 4.0 International license.

10.1128/mSphere.00011-20.3 TABLE S2 Summary statistics for multigenerational *H. saguini* genome assembly and annotation. Download Table S2, DOCX file, 0.01 MB. Copyright © 2020 Mannion et al. 2020 Mannion et al. This content is distributed under the terms of the Creative Commons Attribution 4.0 International license.

10.1128/mSphere.00011-20.4 TABLE S3 Variant sequences identified between the multigenerational genomes. Download Table S3, XLSX file, 0.2 MB. Copyright © 2020 Mannion et al. 2020 Mannion et al. This content is distributed under the terms of the Creative Commons Attribution 4.0 International license.

Based on functional and metabolic predictions, variant genes appeared to affect the interaction of *H. saguini* with its environment, as evidenced by mutations primarily occurring in two-component systems, such as chemotaxis, motility, and nutrient metabolism genes (Fig. 2D). Previous metabolic reconstructions predicted that *H. saguini* is dependent on amino/organic acid precursors and *de novo* biosynthetic pathways to meet carbon and nitrogen demands that fuel energetic needs (27). Alterations in chemotaxis and subsequent metabolism suggest that *H. saguini* may have adapted to the murine host and/or chronic inflammatory environment. Mutations were also detected in virulence factor genes associated with the biosynthesis and modification of lipopolysaccharide, lipooligosaccharides, and the polysaccharide capsule (Table S3). These mutations were often insertions and/or deletions in polynucleotide tracts that caused frameshifts and represent potential phase-variable genes. Mutations in phase-variable surface-associated genes, as described above, have been reported for H. pylori and C. jejuni and have been proposed to enable rapid adaptation to new or changing environments by altering pathogen-host cell interactions at the gastrointestinal mucosal/epithelial surface and antigenicity/detection by the host immune system (18, 21–23, 26, 28–33). The accumulation of gene variants indicated that *H. saguini* undergoes multigenerational microevolutions that may facilitate colonization and survival adaptations in its host. We hypothesized that multigenerational passage of *H. saguini* in germfree IL-10^−/−^ mice yielded gene variants that would enhance its colonization in SPF IL-10^−/−^ murine hosts. Therefore, SPF IL-10^−/−^ mice were inoculated with the *H. saguini* F4 isolate. However, this strain failed to colonize (data not shown), possibly because the murine microbiota precluded *H. saguini* colonization, which was similar to our previous study attempting to infect SPF IL-10^−/−^ mice with this organism (14).

### Conclusion/summary.

Here, we show that *H. saguini* infection can naturally transmit and persistently colonize several successive generations of germfree IL-10^−/−^ mice to cause IBD. Given the coprophagic behavior of mice, horizontal transmission via the fecal-oral route is the likely mechanism for transmitting *H. saguini* infection from the dams to offspring. This is the primary mechanism by which EHS infection, such as H. hepaticus, are also transmitted to new hosts. Whole-genome comparative analyses identified host- and generation-dependent variant genes from *H. saguini* isolates, suggesting that IBD-associated microbes may adapt for colonization and survival in chronic inflammatory environments. However, in this study, *in vitro* culturing may have accounted for some genetic changes, although we attempted to minimize this potential by minimally passaging (≤4 times) *H. saguini* isolates before whole-genome sequencing. Future studies will be required to address the functional significance of the variant genes identified in the multigenerational *H. saguini* isolates. Nevertheless, our results support the hypothesis that transmission of specific microbiota (i.e., *H. saguini*) may be important in initiating and maintaining IBD in captive CTTs. In conclusion, the findings of this study warrant future investigations into the role of the microbiome in the etiopathogenesis of multigenerational IBD in other animal models and humans.

### Methods.

Germfree C57BL/6 (B6.129P2-IL-10*tm1Cgn* [IL-10^−/−^]) mice were infected with *H. saguini* MIT 97-6194-4 as previously described (14). Briefly, a male and female breeding pair (F0 generation) received 0.2 ml of fresh inoculum of *H. saguini* by gastric gavage every other day for three doses and then were bred to produce the F1 generation. The infected F1 female was bred with the F0 male to yield F2, and brother-sister mating produced the sequential F3 and F4 generations. Pups were cohoused with the dams for 5 to 6 weeks after weaning. Three cohorts of mice were included as age-matched controls. Colonization with *H. saguini* was confirmed by PCR of feces using the *Helicobacter* genus-specific primer pair C97/C05. Histopathological evaluation, fluorescence *in situ* hybridization (FISH), immunohistochemistry for γ-H2AX, and quantitative PCR (qPCR) for cytokine gene expression were performed as previously described (14). Colonization levels of *H. saguini* in fecal samples were quantified with qPCR targeting the 16s rRNA gene. Genomic copies were calculated from a standard curve of *H. saguini* genomic DNA in which 1 g of *H. saguini* genomic DNA (∼2.7 Mb) equals ∼3.43 × 10^14^ molecular copies (assuming 1 bp equals ∼650 g/mol and Avogadro’s number is ∼6.02 × 10^23^ molecules/mol). The levels of eukaryotic 18S rRNA from mouse DNA were determined using qPCR. Colonization levels were then normalized for each sample by reporting genomic copies of *H. saguini* per μg of mouse 18S rRNA. Cecal and colonic lesion scores, cytokine mRNA expression, *H. saguini* colonization levels, and γ-H2AX expression were analyzed using the Mann-Whitney U nonparametric test. Statistical analyses were performed using GraphPad Prism, version 5.0 (GraphPad Software, Inc., La Jolla, CA). Results were considered significant at a *P* value of <0.05.

*H. saguini* was cultured from fecal samples and intestinal samples as described previously (14). Isolates were repassaged ≤4 times to minimize potential genetic or phenotypic changes. Purified genomic DNA was sequenced by Illumina MiSeq (34) and Pacific Biosciences RSII (35). Spades version 3.10.1 was used for *de novo* hybrid assembly with the MiSeq reads and the PacBio filtered subreads as long-read scaffolds (36). Resulting contigs were annotated by RAST hosted by PATRIC (37). Sequence variants were identified using the Variation Analysis Service hosted by PATRIC with the BWA-mem aligner and FreeBayes single nucleotide polymorphism (SNP) caller. Further gene annotations and analyses for metabolic function and virulence factors were performed as previously described (27).

### Ethics approval for animal studies.

The protocols for use of animals were approved by the Committee on Animal Care of the Massachusetts Institute of Technology.

### Data availability.

All data and materials generated or analyzed during this study are included in this published article and its supplemental material files or are available from the corresponding author(s) upon request. DNA sequence and assembled genome data have been deposited in NCBI under the BioProject accession no. PRJNA449717.
