# Supplementary figures and images for: Natural Transmission of Helicobacter saguini Causes Multigenerational Inflammatory Bowel Disease in C57/129 IL-10−/− Mice
Source: mSphere. 2020 Mar 25;5(2):e00011-20. doi: 10.1128/mSphere.00011-20 (PMC7096620; doi:10.1128/mSphere.00011-20)

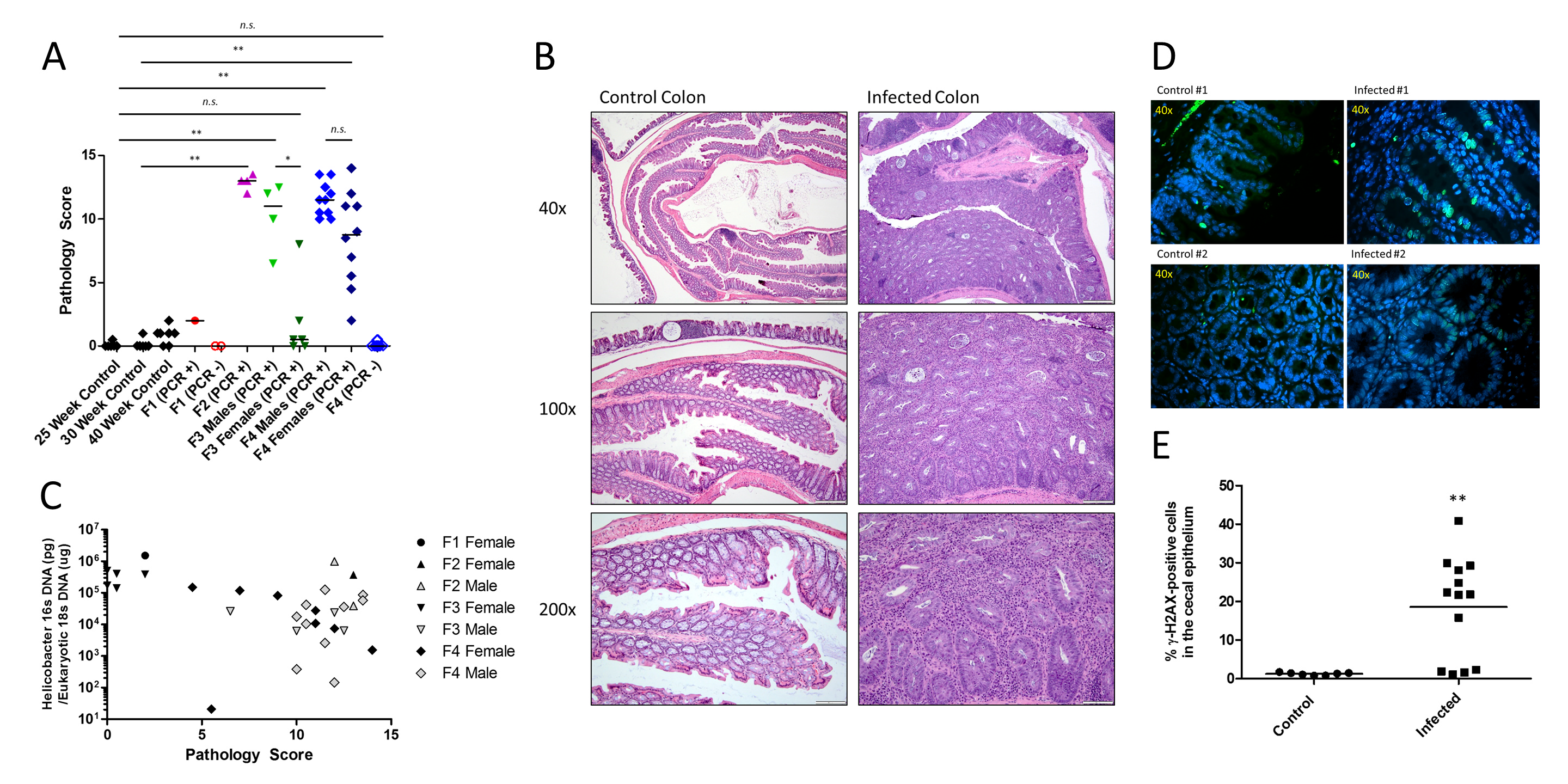

Supplement: FIG S1 [file mSphere.00011-20-sf001.tif]
