# Supplementary material for: Natural Transmission of Helicobacter saguini Causes Multigenerational Inflammatory Bowel Disease in C57/129 IL-10−/− Mice
Source: mSphere. 2020 Mar 25;5(2):e00011-20. doi: 10.1128/mSphere.00011-20 (PMC7096620; doi:10.1128/mSphere.00011-20)
Supplement: TABLE S1 [file mSphere.00011-20-st001.docx]

| **Supplemental Table 1: Sequencing Statistics** | | | | | |
| --- | --- | --- | --- | --- | --- |
| **Genome** | **Sequencing Technology** | **Number of Reads** | **Base Pairs from Reads** | **Genome Size (bp)** | **Estimated Coverage^a^** |
| **F0** | Illumina MiSeq | 1,254,008 | 276,401,984 | 2,756,771 | 139.8 |
|  | PacBio RS II | 17,858 | 108,869,150 |  |  |
| **F2** | Illumina MiSeq | 1,930,538 | 409,057,685 | 2,752,353 | 347.8 |
|  | PacBio RS II | 65,383 | 548,138,798 |  |  |
| **F3** | Illumina MiSeq | 2,638,746 | 497,949,770 | 2,751,696 | 331.8 |
|  | PacBio RS II | 56,912 | 414,976,183 |  |  |
| **F4** | Illumina MiSeq | 2,877,014 | 615,203,467 | 2,752,884 | 338.0 |
|  | PacBio RS II | 42,334 | 315,304,693 |  |  |
| a, Estimated Coverage = Base Pairs from Reads / Genome Size | | | | | |
