# Supplementary material for: Natural Transmission of Helicobacter saguini Causes Multigenerational Inflammatory Bowel Disease in C57/129 IL-10−/− Mice
Source: mSphere. 2020 Mar 25;5(2):e00011-20. doi: 10.1128/mSphere.00011-20 (PMC7096620; doi:10.1128/mSphere.00011-20)
Supplement: TABLE S2 [file mSphere.00011-20-st002.docx]

| **Supplemental Table 2: Genome Annotation Statistics** | | | | | | | |
| --- | --- | --- | --- | --- | --- | --- | --- |
| **Genome** | **Genome Length (bp)** | **Contigs** | **G+C% Content** | **Protein Coding Sequences (CDS)** | **tRNA** | **rRNA** | **GenBank Accession** |
| **F0** | 2,756,771 | 2 | 34.8 | 2,626 | 37 | 4 | QBIX00000000 |
| **F2** | 2,752,353 | 2 | 34.8 | 2,631 | 37 | 4 | QBIW00000000 |
| **F3** | 2,751,696 | 3 | 34.8 | 2,627 | 37 | 4 | QBIV00000000 |
| **F4** | 2,752,884 | 2 | 34.8 | 2,636 | 37 | 4 | QBIU00000000 |
